# Psychological factors and cardiac repolarization instability during anger in implantable cardioverter defibrillator patients

**DOI:** 10.1111/anec.12848

**Published:** 2021-04-03

**Authors:** David S. Krantz, Kristie M. Harris, Heather L. Rogers, Kerry S. Whittaker, Mark C. P. Haigney, Willem J. Kop

**Affiliations:** ^1^ Department of Medical and Clinical Psychology Uniformed Services University of the Health Sciences Bethesda MD USA; ^2^ Department of Internal Medicine Section of Cardiovascular Medicine Yale School of Medicine New Haven CT USA; ^3^ Biocruces Bizkaia Health Research Institute Barakaldo Spain; ^4^ Ikerbasque Basque Foundation for Science Bilbao Spain; ^5^ Research Facilitation Laboratory – Army Analytics Group Monterey CA USA; ^6^ Division of Cardiology Department of Medicine Military Cardiovascular Outcomes Research (MiCOR) Uniformed Services University of the Health Sciences Bethesda MD USA; ^7^ Department of Medical Psychology and Neuropsychology Tilburg University Tilburg The Netherlands

**Keywords:** anger, arrhythmia, QT variability index, repolarization instability

## Abstract

**Background:**

Evidence indicates that emotions such as anger are associated with increased incidence of sudden cardiac death, but the biological mechanisms remain unclear. We tested the hypothesis that, in patients with sudden death vulnerability, anger would be associated with arrhythmic vulnerability, indexed by cardiac repolarization instability.

**Methods:**

Patients with coronary artery disease (CAD) and an implantable cardioverter defibrillator (ICD; *n* = 41) and healthy controls (*n* = 26) gave an anger‐inducing speech (anger recall), rated their current (state) anger, and completed measures of trait (chronic) levels of Anger and Hostility. Repolarization instability was measured using QT Variability Index (QTVI) at resting baseline and during anger recall using continuous ECG.

**Results:**

ICD patients had significantly higher QTVI at baseline and during anger recall compared with controls, indicating greater arrhythmic vulnerability overall. QTVI increased from baseline to anger recall to a similar extent in both groups. In ICD patients but not controls, during anger recall, self‐rated anger was related to QTVI (*r* = .44, *p* = .007). Trait (chronic) Anger Expression (*r* = .26, *p* = .04), Anger Control (*r* = −.26, *p* = .04), and Hostility (*r* = .25, *p* = .05) were each associated with the change in QTVI from baseline to anger recall (ΔQTVI). Moderation analyses evaluated whether psychological trait associations with ΔQTVI were specific to the ICD group. Results indicated that Hostility scores predicted ΔQTVI from baseline to anger recall in ICD patients (β = 0.07, *p* = .01), but not in controls.

**Conclusions:**

Anger increases repolarization lability, but in patients with CAD and arrhythmic vulnerability, chronic and acute anger interact to trigger cardiac repolarization lability associated with susceptibility to malignant arrhythmias.

## 1 INTRODUCTION

Intense emotion, especially anger, can trigger daily life ischemia, malignant arrhythmias, and myocardial infarction in patients with coronary artery disease (CAD) (Chida & Steptoe, 2009; Gabbay et al., 1996; Lampert et al., 2002; Pimple et al., 2015). Chronic (trait) anger‐related characteristics (Carney & Freedland, 2017; Cohen et al., 2015; Krantz & Burg, 2014; Lampert, 2016; Steptoe & Kivimäki, 2013; Wawrzyniak et al., 2015; Williams et al., 2000) and acute anger episodes (Chida & Steptoe, 2009; Mostofsky et al., 2014; Pimple et al., 2015) have been associated with the development and manifestations of CAD, including myocardial infarction (Mostofsky et al., 2014) and ischemia, especially among individuals with left ventricular (LV) dysfunction (Akinboboye et al., 2005; Wawrzyniak et al., 2015). Acute anger episodes can also increase cardiac electrical instability in vulnerable patients (Kop et al., 2004; Lampert et al., 2002; Lampert et al., 2007) and may contribute to the development of malignant arrhythmias (Lampert et al., 2007; Tomaselli et al., 1994).

Increased beat‐to‐beat variability in the QT interval (QT Variability Index or QTVI), a measure of cardiac repolarization lability, is also predictive of increased risk of cardiac arrhythmias and sudden death (Atiga et al., 1998; Berger et al., 1997; Dobson et al., 2011; Haigney et al., 2004; Piccirillo et al., 2007) and is an independent risk marker for, and predictor of, ventricular tachycardia and ventricular fibrillation (Haigney et al., 2004). Little is known about whether chronic anger traits and acute anger responses have effects on the magnitude of repolarization instability as indicated in QT variability, and whether these effects would be limited to patients with increased risk of arrhythmia and sudden death. Therefore, the purpose of this study was to evaluate the link between chronic anger and acute anger reactions and repolarization lability as measured via QT variability index (QTVI). It was hypothesized that both chronic anger traits and anger induced by mental stress would be related to increased QT variability in patients with CAD and arrhythmic vulnerability.

## 2 METHODS

Patients with CAD and implantable cardioverter defibrillators (ICDs) were enrolled because of their known propensity for malignant arrhythmias. Recruitment occurred at 3 medical centers (Arrhythmia Associates, Fairfax VA; Veterans Affairs Medical Center, Washington DC; and St. Francis Hospital, Roslyn NY) as part of the Triggers of Arrhythmia in Defibrillator (TRIAD) study (Haigney et al., 2009; Kop et al., 2004). CAD was documented via angiogram or history of myocardial infarction (MI). Exclusion criteria were atrioventricular conduction defects, left bundle branch block, chronic atrial fibrillation, myocardial infarction *<1*‐month, unstable angina, New York Heart Association class IV congestive heart failure, critical valve pathology, primary cardiomyopathy, use of amiodarone, and age >80 years. For comparison purposes, healthy controls with <5% likelihood of CAD (Rozanski et al., 1984) and no evidence of electrocardiogram (ECG) abnormalities were also tested.

A total of 85 participants were initially enrolled in the TRIAD study (Haigney et al., 2009; Kop et al., 2004). Sixteen ICD patients and 2 healthy controls were excluded due to the noncompletion of trait measures (*n* = 13) or QTVI not being obtained (*n* = 5), resulting in a sample size of 67 (*n* = 41 ICD patients and *n* = 26 healthy controls). Excluded participants were more likely to be nonwhite (*x* ^2^(1) = 4.46, *p* = .04) but did not differ from sample participants in other demographic or baseline characteristics.

To optimize the ECG assessment of QTVI, calcium antagonists and ACE inhibitors were withheld for 24 hr and long‐acting nitrates withheld for 6 hr. Beta‐blockers were withheld for >36 hr in 7 patients, 7 patients were not on beta‐blockers as part of their medical management, and 27 did not discontinue beta‐blockers. Patients tested on beta‐blockers (*n* = 27) had similar QTVI at rest (*p* = .89) and during anger recall testing (*p* = .10) compared with patients tested off beta‐blockers (*n* = 14). This study conformed to the US Federal Policy for the Protection of Human Subjects and was approved by the Institutional Review Boards at the participating institutions. All participants provided written informed consent.

As part of the larger study, participants completed a 2‐day laboratory mental stress procedure that included testing with the anger recall task. After a 15‐min resting baseline, participants were asked to recall a recent incident in which they felt irritated, frustrated, angry, or upset and instructed to deliver a 4‐min speech about this anger‐provoking situation to the research team. Anger recall is a potent task capable of producing significant hemodynamic responses and impairments in ventricular function and myocardial ischemia in CAD patients (Jain et al., 2001; Jiang et al., 2013; Steptoe & Kivimäki, 2013). Participants rated their current (state) level of anger at baseline and during anger recall task using a Likert scale from 0 = not at all to 7 = very much.

During rest and anger recall, blood pressure was obtained every 60 s, and continuous digitized ECGs were obtained during rest and anger recall. Recording and digitalization of ECG occurred at 1,000 Hz with 16‐bit resolution using the CH2000 (Cambridge Heart) with high‐resolution silver–silver chloride electrodes for noise reduction. Data were exported for blinded off‐line QT variability analyses by a trained reader using the method developed by Berger and colleagues (Berger et al., 1997). Each QT interval and heart rate value were measured over equal time periods obtained from the lead with the best visualization of the QT interval (generally lead III). A QT interval template is selected from a representative beat and compared with each subsequent beat and stretched or compressed in time to achieve a high degree of fit. The stretch factor for each beat is used to derive a QT and heart rate time series for the epoch. Beats preceding and following ectopy, and all artifacts were excluded and removed prior to analyses. QT variability index (QTVI) was calculated as the log ratio of normalized QT variability (QTVN; QT interval variance divided by QT interval mean‐squared) to normalized HR variability (heart rate variance divided by heart rate variance mean‐squared). QTVI scores are quantified as negative numbers, with QTVI indicating higher arrhythmic vulnerability having a negative value closer to 0 (i.e., smaller negative number), and QTVI indicating lower vulnerability having a negative value further from 0 (i.e., larger negative number).

Prior to the study, participants completed self‐report measures of trait (chronic) Anger and Hostility. The 24‐item Spielberger Anger Expression Scale is comprised of 3 independent subscales (Spielberger et al., 1985). Anger Suppression and Anger Expression measure an individual's general tendency to suppress anger (e.g., “I boil inside but don't show it”) or express anger toward other people or objects in the environment (e.g., “I strike out at whatever infuriates me”), respectively, whereas higher scores on Anger Control are associated with less frequent experiences of anger or aggressive behavior (e.g., “I keep my cool”). Respondents rate how characteristic each item is for them from 1 (almost never) to 4 (almost always) and subscale scores range from 8 to 32. Internal consistency reliability in the present study was acceptable for each subscale (Cronbach's α between .66 and .82). Participants also completed the 50‐item Cook‐Medley Hostility Scale (Cook & Medley, 1954; Williams et al., 1980). Respondents rate each statement as true or false as applied to them, with higher scores indicating greater hostility. The Hostility scale has good psychometric properties (Cook & Medley, 1954; Williams et al., 1980).

Data are presented as mean ± standard deviation (*SD*) or frequencies (N) and percentages (%), and *t* tests and chi‐squared tests used to evaluate demographics. Differences between ICD patients and healthy controls in trait variables (Anger Suppression, Anger Expression, Anger Control, and Hostility), and baseline and anger recall values of QTVI, hemodynamics, and anger ratings were evaluated with analysis of covariance (ANCOVA), adjusted for age, sex, and race (white vs. nonwhite). Repeated measures ANCOVA was used to evaluate group differences in QTVI, hemodynamics, and self‐rated anger rating levels during baseline and anger recall, with time as a within‐subjects factor (baseline vs. anger recall), group (ICD patients vs. controls) as a between‐subjects factor, and covariates of age, sex, and race. Paired samples *t* tests were used to examine changes from baseline to anger recall within each group of QTVI, hemodynamics, and state anger ratings. Effect size was calculated using partial eta squared (η_p_^2^) and Cohen's d.

Pearson correlations examined associations of trait variables (Anger Suppression, Anger Expression, Anger Control, Hostility) and state anger self‐ratings (during anger recall and Δ from baseline to anger recall) with QTVI (baseline, anger recall, and Δ from baseline to anger recall) in the total sample and within each group. To determine whether associations between anger/hostility traits and ΔQTVI were moderated by group membership (ICD patient vs. controls), significant correlations were evaluated further using linear regressions and the PROCESS V3.0 macro for SPSS (Hayes, 2018). Models were adjusted for covariates to predict ΔQTVI from anger/hostility traits, ICD group, and the Anger/Hostility by group interaction. Analyses used SPSS Statistics for Mac v.26 (IBM Corp.) and utilized a 2‐tailed alpha‐level of *p* < .05.

## 3 RESULTS

Sample demographics and clinical characteristics of the ICD patients are shown in Table 1. Compared with Controls, ICD patients were older (*t*(65) = −2.23, *p* = .03) and more likely to be male (*x* ^2^(1) = 12.21, *p* <.001). Participants in both groups were mostly white and non‐Hispanic.

**TABLE 1:** Group demographics and ICD patient clinical characteristics

	Healthy Controls (*n* = 26)	ICD Patients (*n* = 41)
Age, years	56.0 ± 11.6	61.8 ± 9.5*
Sex (male)	16 (61.5)	39 (95.1)*
Race (White, non‐Hispanic)	23 (88.5)	38 (92.7)
Number of diseased vessels^a^		
1	–	7 (17.1)
2	–	15 (36.6)
3	–	18 (43.9)
Ejection fraction, %	–	36.1 ± 12.1
Medical history		
Coronary angioplasty	–	23 (56.1)
Coronary artery bypass graft surgery	–	22 (53.7)
Myocardial infarction	–	35 (85.4)
Cardiomyopathy	–	15 (36.6)
Hypertension	–	27 (65.9)
Diabetes mellitus	–	12 (29.3)
Medications		
Beta‐blocker	–	34 (82.9)
Calcium channel blocker	–	3 (7.3)
ACE inhibitor	–	23 (56.1)
Anti‐arrhythmic agent	–	4 (9.8)

Note

Values are mean ± *SD* or *n* (%).

^a^ Data not available for 1 participant.

* *p* < .05 for independent samples *t* test or chi‐square comparisons between groups

Table 2 displays group means for QTVI, its components, hemodynamics, state anger ratings, and trait variables, with between‐group comparisons adjusted for age, sex, and race. QTVI increased from baseline to anger recall within both groups (*p* < .05; Table 2), but ICD patients had significantly higher QTVI at baseline (*F*(1,62) = 11.04, *p* = .001, η_p_^2^ = 0.15) and during anger recall (*F*(1,58) = 16.05, *p* < .001, η_p_^2^ = 0.22) compared with healthy controls, indicating higher arrhythmic vulnerability. These group differences were primarily explained by differences in the QTVI numerator (i.e., QTV unadjusted for HR power), which was also higher in ICD patients than controls at both baseline (*F*(1,62) = 3.99, *p* = .05, η_p_^2^ = 0.06) and during anger recall (*F*(1,58) = 8.56, *p* = .005, η_p_^2^ = 0.13), and not by changes in the heart rate‐based QTVI denominator. However, the groups did not differ in the standard deviation of NN intervals (SDNN), a time‐domain measure of heart rate variability, at rest or during anger recall, and anger recall did not provoke a significant response in SDNN in either group (all *p* > .09). There were no group differences in blood pressure or heart rate at rest or during anger recall, and within both groups, blood pressure and heart rate significantly increased from baseline to anger recall (all *p* < .001).

**TABLE 2:** Means and comparisons of QTVI variability components, hemodynamics, state anger, and anger/hostility traits

	Within‐group comparisons						Between‐group comparisons		
	Healthy Controls (*n* = 26)			ICD Patients (*n* = 41)			ICD Patients versus Controls (*p*‐values)		
	Baseline	Anger Recall	Δ	Baseline	Anger Recall	Δ	Baseline	Anger Recall	Δ
Repolarization instability									
QTVI	−1.6 ± 0.3	−1.3 ± 0.3*	0.3 ± 0.4	−1.0 ± 0.7	−0.7 ± 0.6*	0.3 ± 0.7	0.001	<0.001	0.85
QTVI Numerator	0.06 ± 0.05	0.16 ± 0.13	0.09 ± 0.11	0.22 ± 0.36	0.41 ± 0.72	0.18 ± 0.75	0.05	0.005	0.08
QTVI Denominator	2.4 ± 2.5	3.0 ± 2.4	0.6 ± 1.4	2.8 ± 4.2	1.6 ± 2.0	−1.0 ± 4.2	0.23	0.16	0.10
SDNN, ms	44.0 ± 27.1	48.1 ± 22.4	4.05 ± 20.2	47.8 ± 43.6	37.0 ± 25.1	−9.4 ± 33.2	0.28	0.28	0.08
Hemodynamics									
Systolic blood pressure, mmHg	124 ± 15	148 ± 29*	24 ± 18	136 ± 21	166 ± 22*	30 ± 16	0.20	0.32	0.92
Diastolic blood pressure, mmHg	76 ± 8	90 ± 10*	15 ± 8	78 ± 11	94 ± 12*	15 ± 6	0.43	0.68	0.71
Heart rate, bpm	69 ± 9	78 ± 11*	8 ± 7	64 ± 11	73 ± 13*	9 ± 7	0.07	0.22	0.66
Current (State) anger likert rating	1.1 ± 0.4	5.1 ± 1.2*	4.0 ± 1.3	1.4 ± 1.3	4.7 ± 1.6*	3.2 ± 1.7	0.32	0.85	0.38
Trait variables									
Anger expression	12.6 ± 2.9	–	–	13.8 ± 2.8	–	–	0.008	–	–
Anger suppression	15.0 ± 2.9	–	–	15.4 ± 3.2	–	–	0.80	–	–
Anger control	24.6 ± 4.6	–	–	24.2 ± 4.0	–	–	0.21	–	–
Hostility	11.9 ± 6.1	–	–	13.7 ± 5.4	–	–	0.28	–	–

Note

All values represent mean ± *SD*; Δ = change from baseline to anger recall; QTVI = QT Variability Index; SDNN = standard deviation of NN intervals; Within‐group comparisons are tested using paired samples *t* tests with *p < .05. For analyses of between‐group comparisons, baseline and anger recall values are tested using ANCOVA, and Δ values are tested using repeated measures ANCOVA. All between‐subjects comparisons adjust for age, sex, and race.

Resting baseline self‐ratings of current (state) anger were low and comparable among groups and significantly increased within both groups during anger recall (Controls: (*t*(25) = −15.41, *p* < .001, Cohen's d = 3.02; ICD patients: *t*(40) = −12.44, *p* < .001, Cohen's d = 1.94). Adjusting for any differences in age, sex, and race, trait variables were similar among ICD patients and healthy controls (Table 2), except for Anger Expression, which was higher in the ICD group (*F*(1,62) = 7.59, *p* = .008, η_p_^2^ = 0.11).

**FIGURE 1:**
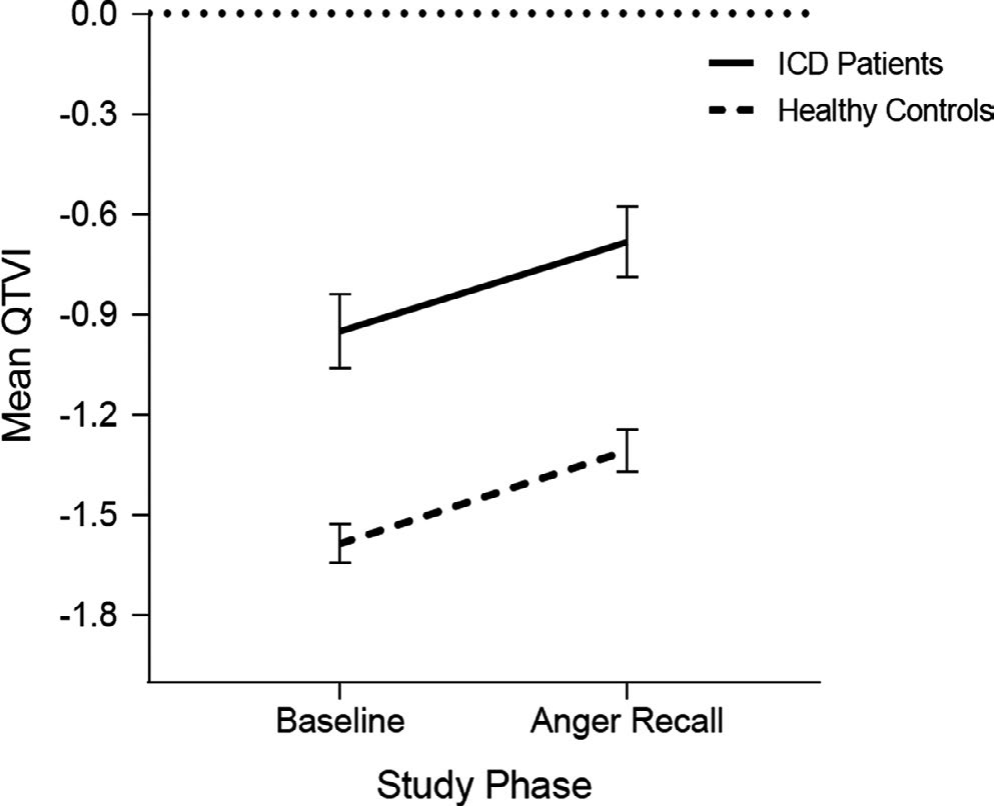
QTVI at rest and during anger recall in ICD Patients and Healthy Controls. QTVI values closer to 0 (i.e., smaller negative number) are indicative of higher arrhythmic vulnerability, while values further from 0 (i.e., larger negative number) indicate lower arrhythmic vulnerability

Although levels of QTVI were consistently elevated in ICD patients compared with controls, there was no time by ICD group interaction (Figure 1), and the magnitude of QTVI change(Δ) from baseline to anger recall was comparable in both groups (*p* = .85), with a similar pattern observed for the Δ in QT numerator, QT denominator, and SDNN (all *p* > .08). The magnitude of systolic and diastolic blood pressure and heart rate Δ from baseline to anger recall was also comparable among ICD patients and controls (all *p* > .66). ICD patients and controls reported similar increases in state anger ratings in response to anger recall (*p* = .38).

In the overall sample (*n* = 67), trait Anger and Hostility measures and self‐rated anger were not associated with QTVI at baseline or during anger recall (all *r* < .20 and *p* > .11). Similarly, Δ in self‐rated anger in the overall sample from baseline to anger recall were not associated with the magnitude of ΔQTVI from baseline to anger recall (*p* = .38). However, there were significant associations with ΔQTVI with Anger Expression (*r* = .26, *p* = .04), Anger Control (*r* = −.26, *p* = .04), and Hostility (*r* = .25, *p* = .05), but not with Anger Suppression (*p* = .39). In ICD patients, but not in controls, magnitude of self‐rated anger during the anger recall task was related to QTVI (*r* = .44, *p* = .007).

**FIGURE 2:**
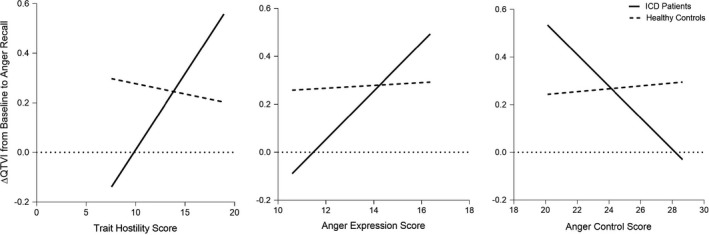
Associations of Anger/Hostility traits and ΔQTVI from baseline to anger recall are moderated by (conditional upon) Group membership (ICD patient vs. Controls), with higher ΔQTVI, indicating increasing arrhythmic vulnerability, during anger in ICD patients. Plots depict calculated regression slopes. ΔQTVI = change from resting baseline to anger recall, with higher ΔQTVI indicating increasing arrhythmic vulnerability. QTVI values closer to 0 (i.e., smaller negative number) indicate higher arrhythmic vulnerability, and values further from 0 (i.e., larger negative number) indicate lower arrhythmic vulnerability

We next used covariate‐adjusted models of simple moderation (PROCESS model 1; Hayes, 2018) to determine whether these significant relationships of QTVI changes to anger/hostility traits were conditional on (i.e., dependent on) group membership and occurred in ICD patients but not healthy controls. There was a significant group interaction with Hostility scores (β = 0.07, *p* = .01), with increasing Hostility associated with ΔQTVI from baseline to anger recall only in ICD patients and not controls (Figure 2), with a similar pattern for the interaction of ICD group with decreasing Anger Control (β = −0.07, *p* = .06). The ICD group by Anger Expression interaction also looked similar but was not significant in this sample (β = 0.10, *p* = .10).

## 4 DISCUSSION

Acute anger provocation during mental stress was associated with an increase in the QT Variability Index marker of repolarization instability. However, magnitude of anger‐induced increases in the QT Variability Index is associated with trait (chronic) anger only in CAD patients with arrhythmic vulnerability, but not in healthy controls. QTVI has been shown to predict CV death and ICD discharges (Atiga et al., 1998; Berger et al., 1997; Dobson et al., 2011; Haigney et al., 2004; Piccirillo et al., 2007). Thus, in the context of previously existing CAD and arrhythmic vulnerability, psychological characteristics associated with anger and hostility are indicative of susceptibility to cardiac repolarization lability and heightened susceptibility to malignant arrhythmias during acute anger.

These data are consistent with findings that acute mental stress may precipitate increases in QT Variability (Magrì et al., 2012) and QT dispersion (Hassan et al., 2009; James et al., 2000) in post‐MI and CAD patients. Previous findings with this sample (Kop et al., 2004) and in canines (Kovach et al., 2001), showed that mental stress and anger cause an increase in T‐wave Alternans, another marker of cardiac electrical instability. In vulnerable patients, malignant arrhythmias and markers of arrhythmic vulnerability are also induced by anger (Burg et al., 2004; Lampert et al., 2002; Lampert et al., 2007; Verrier & Mittleman, 1996). Other investigators did not find a relationship of hostility to QT variability at rest in a population of normal controls and in patients with panic disorder, but reported higher QT variability among individuals with panic disorder and depression (Yeragani & Kumar, 2000; Yeragani et al., 2000). However, those studies differ from the present study in both patient population (panic disorder patients vs. ICD patients), and the conditions under which QT variability was measured (at rest vs. changes induced by anger).

Both healthy controls and ICD subjects in the present study manifest an increase in QTVI with anger. However, the mechanisms driving this increase appears to be different in these 2 groups. QTVI is a ratio function, and an increase in QTVI may be driven by an increase in the numerator (i.e., increase in the normalized QT variability) or by a decrease in the denominator (i.e., a reduction in heart rate variability). ICD patients and controls both increased in the numerator function, or QTVN, but the average value of normalized QT variability during anger recall was higher (*p* < .005) in ICD patients. This suggests that, compared with healthy controls, anger increases repolarization instability out of proportion to the effects on autonomic tone.

### 4.1 Study limitations

The present sample was predominantly male, selected based on the presence of coronary disease, and criteria for ICD implantation have changed over time based on the MADIT II and subsequent studies (Kusumoto et al., 2018; Moss et al., 2002). Therefore, the results may not generalize to the larger and more diverse population of patients with and without CAD receiving ICDs today. The mean QTVI values of our ICD population are comparable; however, to those we reported in GISSI‐HF (Dobson et al., 2011). Similarly, although every effort was made to withhold medications from the ICD patients in the study, many patients were on medications that may have influenced the present results. Lastly, the present study was designed to provide information regarding dynamic changes in cardiac repolarization that occur during mental stress, rather than for purposes to risk stratification.

Increased QTVI predicts sudden death (Dobson et al., 2011; Piccirillo et al., 2007) and ventricular tachycardia or fibrillation (Haigney et al., 2004). An increase in QTVI indicates that the QT interval is varying out of proportion to the heart rate, implying loss of “repolarization reserve,” the capacity of the myocardium to regulate excitability in response to changing autonomic tone. Conditions manifesting reduced repolarization reserve are associated with an increased risk of re‐entrant arrhythmias such as polymorphic ventricular tachycardia or fibrillation.

The present results add to a body of knowledge regarding dynamic changes in cardiac repolarization that occur during mental stress and suggest that, in vulnerable patients, abnormalities in ventricular repolarization and cardiac repolarization lability may be one mechanism linking acute anger to arrhythmias and/or sudden cardiac death (Atiga et al., 1998; Burg et al., 2004; Dobson et al., 2011; Lampert et al., 2002). Furthermore, in patients with previously existing CAD and arrhythmic vulnerability, chronic anger traits potentiate the effects of acute anger and may be particularly important triggers of cardiac repolarization lability and susceptibility to malignant arrhythmias.

## ETHICAL APPROVAL

This study conforms to the US Federal policy for protection of human subjects and was reviewed and approved by Institutional Review Boards at the Uniformed Services University, St. Francis Hospital, Roslyn NY, VA Medical Center, Washington DC, and INOVA Hospital, Fairfax, VA.

## CONFLICTS OF INTEREST

The authors have stated explicitly that there are no conflicts of interest in connection with this article. This research was reviewed and approved by institutional review boards at the Uniformed Services University, St. Francis Hospital, Roslyn, NY, and INOVA Hospital in Fairfax, VA

## Data Availability

Data are maintained by Dr. David Krantz, Study Principal Investigator, at the Uniformed Services University of the Health Sciences. Data are available from Dr. David Krantz (david.krantz@usuhs.edu) upon request.
